# The Effects of Probiotics on Cholesterol Levels in Patients With Metabolic Syndrome: A Systematic Review

**DOI:** 10.7759/cureus.37567

**Published:** 2023-04-14

**Authors:** Elina S Momin, Asma A Khan, Tejasvi Kashyap, Muhammad Ahad Pervaiz, Aqsa Akram, Vijayalakshmi Mannan, Muhammad Sanusi, Abeer O Elshaikh

**Affiliations:** 1 Internal Medicine, California Institute of Behavioral Neurosciences & Psychology, Fairfield, USA; 2 Medical School, California Institute of Behavioral Neurosciences & Psychology, Fairfield, USA; 3 General Practice, California Institute of Behavioral Neurosciences & Psychology, Fairfield, USA; 4 Urology, California Institute of Behavioral Neurosciences & Psychology, Fairfield, USA; 5 Internal Medicine, Shenyang Medical College, Shenyang, CHN; 6 Internal Medicine/Family Medicine, California Institute of Behavioral Neurosciences & Psychology, Fairfield, USA

**Keywords:** very low density lipoprotein, lactobacillus, yogurt, low density lipoprotein-cholesterol, metabolic syndrome, cholesterol, probiotics

## Abstract

The prevalence of metabolic syndrome has been increasing over the past few years, especially in the United States. As a result, it increases the risk of heart disease, stroke, and diabetes mellitus, thus causing significant health issues. Probiotics have been studied to have effects on maintaining blood cholesterol levels by altering the gut microbiota. This systematic review aims to find the effects that probiotics would have on lipid levels when given to patients with metabolic syndrome. In total, articles collected from PubMed, Google Scholar, and ScienceDirect were analysed. The results of the majority of the studies revealed that probiotics have some significant effects on cholesterol levels. It has shown a reduction in triglycerides and lower-density lipoprotein (LDL), thereby decreasing cholesterol levels in the blood. However, further investigations must be carried out so in order to create a more detailed and specific explanation of the effects and mechanisms of probiotics on maintaining cholesterol levels in the blood.

## Introduction and background

Metabolic syndrome refers to a group of five conditions that can lead to heart disease, diabetes, stroke, and other health problems [1]. Metabolic syndrome is diagnosed when someone has three or more risk factors: high blood glucose, low levels of high-density cholesterol (HDL) in the blood, high levels of triglycerides in the blood, large waist circumference (WC), and high blood pressure [1]. Metabolic syndrome increases the risk of cerebrovascular and cardiovascular risk two-fold [2]. According to the National Health and Nutrition Examination Survey in the US, weighted metabolic syndrome prevalence has steadily increased from 32.5% in 2011-2012, 34.6% in 2013-2014, and 36.9% in 2015-2016 [3]. The predominant underlying risk factors for the syndrome appear to be abdominal obesity, insulin resistance and physical inactivity, ageing, and hormonal imbalance [4]. Its management includes the controlling of these underlying risk factors [5]; for instance, obese patients need their calorie intake reduced by 500-1000 calories per day to reduce their weight by about 7-10% over 6-12 months, followed by long-term behaviour modification and maintenance of increased physical activity with a balanced diet [5]. The blood pressure should be reduced to less than 140/90 mmHg through lifestyle or medication [6]. For patients with increased fasting blood glucose or diabetes, glucose levels should be maintained with lifestyle changes and, if required, drug therapies such as oral hypoglycemic agents and insulin [6]. The goal should be to have a haemoglobin A1 c of less than 7% [6].

Cholesterol is a lipophilic molecule that is essential for human life [7]. While cholesterol is vital as it helps the body build healthy cells, too much of it can increase the risk of heart disease, stroke, and pancreatitis [8]. Patients with metabolic syndrome should have their blood cholesterol checked through lipid panels (total cholesterol (TC), HDL, low-density cholesterol (LDL), and triglycerides) [9]. In patients with increased cholesterol levels, usually moderate to high-intensity statin therapy is given first because elevated LDL should also be aggressively managed with the goal of dropping it by 50%, especially if the atherosclerotic cardiovascular disease (ASCVD) risk score is more than 7.5%, which establishes patients' 10-year ASCVD risk [10].

Probiotics are microorganisms that can exert advantageous health effects on the body by improving gut microbial balance [11]. Recent studies suggest that manipulating the gut microbiota through probiotics could be an effective approach for preventing and managing metabolic syndrome [12] as they modulate colon microflora and immunogenic responses in the gut, improving health [13]. Oral ingestion of probiotic bacteria has been used as a gut microbiota-targeted strategy to fight metabolic syndrome [14]. Sources of probiotics are yoghurt, Greek yoghurt, smoothies, pickled onions, pickled beet, buttermilk, cottage cheese, garlic, apple cider vinegar, etc [15]. Lactobacilli and bifidobacteria are the most common bacteria and are many times related to these useful probiotic effects [16]. Studies have connected these Lactobacillus and Bifidobacterium genera with human gut health and metabolic functions [17]. The use of such orally introduced organisms (probiotics) is a major aim of the concept of functional food [17]. Probiotics decrease plasma LDL and TC in subjects with normal, borderline high, and high cholesterol levels [18]. Probiotics, which contain living strains of bacteria that add to the population of good bacteria in the intestine, are promoted by prebiotics, specialized plant fibres that act as food for probiotics [19].

**Figure 1:**
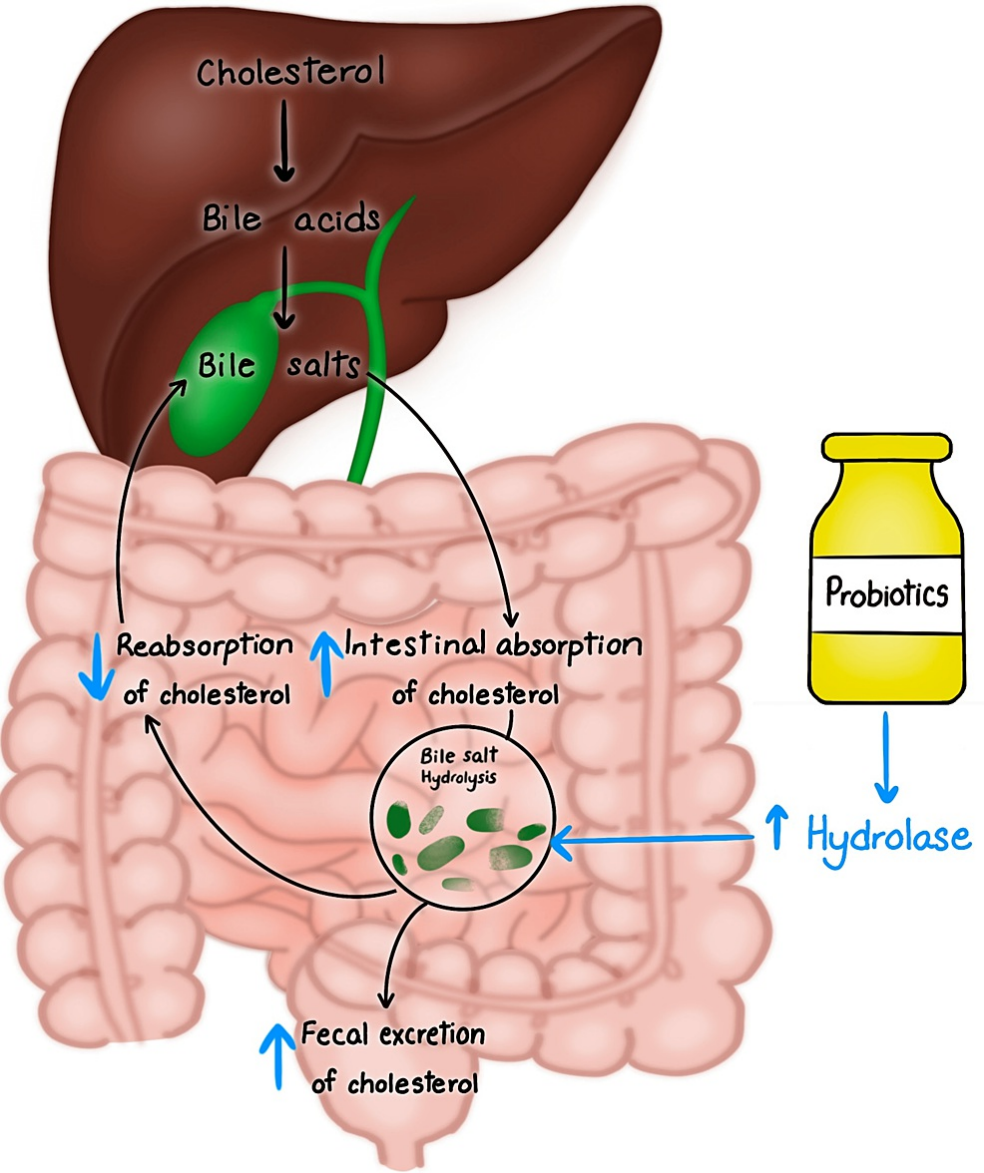
Mechanism of action of probiotics in lowering cholesterol levels. This figure is an original diagram created by one of the co-authors (Tejasvi Kashyap).

Figure 1 demonstrates the mechanism of action of probiotics on bile salt hydrolase in decreasing the blood cholesterol level.

## Review

Method

This systematic review was designed using the Preferred Reporting Items for Systematic Reviews and Meta-Analysis (PRISMA) 2020 guidelines [20].

Sources of data and search strategy

We collected our articles using the PubMed, Google Scholar, and ScienceDirect.com databases. Articles were obtained using medical subject headings (MeSH) and keywords combined with Boolean connectors (AND) in PubMed. 

Table 1 shows the search strategy used in various databases to collect the articles.

**Table 1:** Shows the search strategy for various databases. LDL: lower density lipoprotein

database	Search Strategy	articles
PubMed	Probiotics OR Probiotics flora OR Probiotics microflora OR prebiotics OR synbiotics OR Good bacteria OR ( "Probiotics/metabolism"[Mesh] OR "Probiotics/pharmacology"[Mesh] OR "Probiotics/physiology"[Mesh] OR "Probiotics/therapeutic use" [Mesh] )) AND (CHOLESTEROL OR Lower Density Lipoprotein OR Very Low Density lipoprotein OR LDL cholesterol OR High Density Lipoprotein OR ( "Cholesterol/biosynthesis"[Mesh] OR "Cholesterol/chemical synthesis"[Mesh] OR "Cholesterol/genetics"[Mesh] OR "Cholesterol/metabolism"[Mesh] OR "Cholesterol/physiology"[Mesh] OR "Cholesterol/therapeutic use"[Mesh] OR "Cholesterol/therapy"[Mesh] ))) AND (Metabolic syndrome OR Syndrome X OR insulin resistance syndrome OR dysmetabolic syndrome OR ( "Metabolic Syndrome/diet therapy"[Mesh] OR "Metabolic Syndrome/metabolism"[Mesh] OR "Metabolic Syndrome/physiology"[Mesh] OR "Metabolic Syndrome/physiopathology"[Mesh] OR "Metabolic Syndrome/prevention and control"[Mesh] OR "Metabolic Syndrome/therapy"[Mesh	183
Google Scholar	Probiotics ,cholesterol ,metabolic syndrome.	36
Science Direct	Probiotics, cholesterol, metabolic syndrome.	36

Inclusion and Exclusion Criteria

This systematic review included adult humans. It included texts in the English language with open free access. The articles were selected from the last 10 years. The study included randomised controlled trials, meta-analyses, systematic reviews, and traditional reviews. Animals were excluded from the studies. Non-English language papers and studies involving the paediatric population were excluded from the study.

Risk and quality assessments: Two independent reviewers conducted the risk and quality assessment for each of the seven articles. The Amstar checklist was used for systematic review, the Cochrane risk of bias assessment tool was used for randomised controlled trials, and SANRA, a scale for quality assessment of narrative articles, were used. The studies meeting the criteria of more than 70% for quality and grade were selected for the systematic review.

Data extraction: The final seven included articles were reviewed by two separate independent researchers through the Rayyan software (Rayyan Systems, Cambridge, MA) [21]. Data were extracted and put under the following headings of the first author with the year of publishing of the article, location, study type, type of probiotic used, duration of intervention, and effects on cholesterol levels.

Results

Study Selection

**Figure 2:**
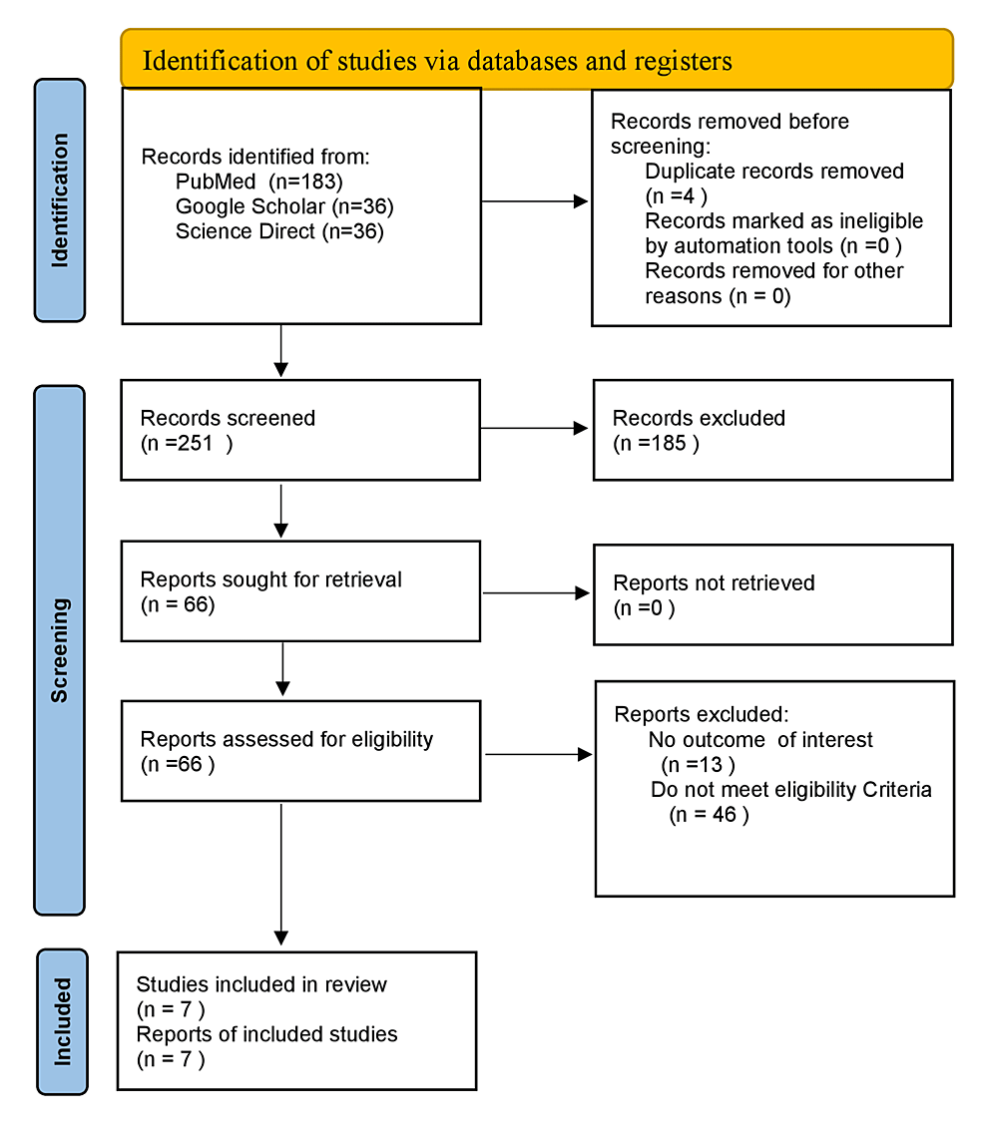
PRISMA flow diagram

For this study, 251 records were obtained from PubMed, ScienceDirect, and Google Scholar. These records were transferred to Rayyan software for systematic review. After undergoing screening by two independent reviewers, 185 articles were excluded for being irrelevant based on abstract and background articles. A total of 66 articles were gathered and were assessed for eligibility, again by two reviewers who read the full text of the remaining 66 articles to check for their eligibility of the articles. Finally, seven articles were included in the studies. A complete PRISMA flow diagram is shown in Figure 2 [20].

Below is Table 2 with baseline characteristics of included studies which gives information on the type of probiotic use, duration of intervention, and its effects on cholesterol levels.

**Table 2:** Baseline characteristics of included studies. TG: triglycerides; LDL: low-density lipoprotein; HDL: high-density lipoprotein; TC: total cholesterol; SMD: standard mean difference; MCID: minimal clinically important difference

	First Author/ Year	Location	Study type	Type of probiotic used	Duration of intervention	Effect on cholesterol levels
1	He et al. [12] 2017	China	Traditional review (Observational study)	*L. acidophilus* and *Bifidobacterium lactis*, in addition to the bacteria in ordinary yogurt containing microencapsulated bile salt hydrolase-active *Lactobacillus reuteri* NCIMB 30242 daily consumption of 200 gm of fermented milk (FM) containing *L. acidophilus* L1, a mixture of organisms (a probiotic mixture) comprised of Bacillus, Saccharomyces, Streptococcus, Clostridium, Lactobacillus, and Candida.	Six weeks, two times per day during six weeks.	Significantly reduced LDL-cholesterol and TC
2	Pan et al. [3] 2021	China	Systematic review	Probiotic therapy	Three weeks	Significantly reduced the TC (SMD = −0.36, 95% CI −0.55, −0.17), LDL cholesterol (SMD = −0.42, 95% CI −0.61, −0.22, but increased the HDL cholesterol (SMD = 0.28, 95% CI.03, 0.52).
3	Dieck et al. 2021 [16]	Germany	Traditional review	Probiotic therapy capsules comprising Bacillus subtilis, 290 mg L-alanyl-L-glutamine, 90 mg curcuma extract (approx. 70–80% curcumin), 90 mg green tea extract (approx. 50% EGCG), 5 mg zinc, 0.56 mg vitamin B6, 20 µg D-biotin, 0.75 µg vitamin B12, four µg vitamin D, 2.4 mg pantothenic acid.	Four weeks	Relatively small but significant reductions of LDL cholesterol and TC levels
4	Arabi et.al. 2022 [2]	Iran and Perth, Australia	Systematic review and meta-analysis	Probiotic therapy		The reduction of LDL cholesterol was higher than the minimal clinically important difference (MCID) for LDL (3.87 mg/dl), which is essential for clinicians to reach treatment goals. Increase in HDL.
5	Dong et al. 2019 [22]	USA and Shanghai	Systematic review and meta-analysis	Probiotic foods or supplements.	Six weeks to 24 weeks	No significant differences in TC and HDL cholesterol, but a significant reduction was found in LDL cholesterol.
6	Cicero et al. 2021 [23]	Italy	Randomised controlled trial	The probiotic formula of L. Plantarum PBS067—DSM 24,937, L. acidophilus PBS066—DSM 24,936 and L. reuteri PBS072.	Two months	Decreased LDL cholesterol, HDL cholesterol, TC and triglycerides.
7	Bernini et al. 2016 [17]	Brazil	Randomised controlled trial	Milk fermented with probiotics	45 days	No significant differences were observed intra- or intergroup for TGs and HDL cholesterol.

Discussion

Role of Probiotics in Cholesterol Reduction

According to a study published by Bernini et al., consuming milk fermented with Lactobacillus plantarum reduced low-density lipoprotein ( LDL), total cholesterol (TC), glucose, and IL-6 [17]. There are mechanisms that show that some probiotic microorganisms can produce hydrolase that can cause the deconjugation of bile acids [17]. Deconjugated bile acids are less effectively absorbed, resulting in increased faecal excretion [17]. Cholesterol coprecipitation with bile acids that are deconjugated leads to reduced solubilization and absorption of lipids from the diet, which can lead to decreased cholesterol [17]. Probiotic usage is associated with increased levels of short-chain fatty acids (SCFA) like propionate, which inhibits hydroxymethylglutaryl coenzyme A reductase (HMGCoA reductase) present in the liver. It is a rate-limiting step of the cholesterol synthesis pathway, and this leads to improved cholesterol metabolism [17]. 

According to another study by He et al., the probiotics elevate bacteria-derived short-chain fatty acids (SCFA), which activate G-protein receptor-43 (GPR-43) on L cells (majorly found in the ileum and large intestine) and trigger the secretion of glucagon-like peptide-1 (GLP-1) and glucagon-like peptide-2 (GLP-2) which play a vital role in lipid metabolism [12]. Probiotics which produce SCFA can elevate SCFA influx in the liver. This causes down-regulation of angiopoietin-like protein 4 (ANGPTL4), which inhibits circulating lipoprotein lipase (LPL) followed by lipid clearance [12]. The downstream target gene of peroxisome proliferator-activated proliferator-activated receptors (PPARs) is ANGPTL4 [12]. PPAR-α has an important role in hepatic fatty acid oxidation, whereas PPAR-γ plays a vital role in adipogenesis, and this is why PPAR agonists are used to treat diabetes and cardiovascular diseases [12]. Bile salt hydrolase present in probiotics causes the deconjugation of bile salts such as conjugated glycodeoxycholic acid and taurodeoxycholic acid [12]. Bile salts are essential in forming micelles that help absorb cholesterol in the intestines, and thus, probiotics, by disrupting micelle formation, decrease cholesterol absorption [12]. When bile acid enters the enterohepatic circulation, conjugated bile acid hydrolase enzymes (hydroxysteroid dehydrogenases) present in probiotics hydrolyze the bile acid and bile salts [12]. This disrupts the enterohepatic circulation of bile acids and thus decreases cholesterol absorption. Another mechanism is strengthening the anti-inflammatory function of probiotics, which improves low-grade inflammation, glucose intolerance, insulin sensitivity and steatosis [12]. The final mechanism is the down-regulation of endocannabinoid (eCB) system responsiveness, which is associated with regulating energy homeostasis and normalising adipogenesis [12].

A study published by Dong et al. indicated that basic mechanisms of the antagonistic effects of probiotics include improvement of the gut barrier function, increased competitive adherence to the mucosa and epithelium, gut microbiota modification, and regulation of the gut-associated lymphoid immune system [22].

A study by Pan et al. shows that probiotics can restore disturbed microbial function by alleviating obesity, blood lipids, and even inflammation in patients [3]. Patients with metabolic syndrome showed a sharp decline in Gram-positive bacteria and an increase in Gram-negative bacteria [3]. Specific Gram-positive bacteria, like bile salt-hydrolyzing Lactobacillus and the reuteri strain, can inhibit lipoprotein lipase, the enzyme responsible for triglyceride hydrolysis, and, therefore, work against calorie uptake from the gut and storage in adipose tissue [3]. Additionally, Gram-positive microbiota (mainly Lactobacillus and Bifidobacterium) could degrade complex plant-derived polysaccharides to SCFAs [3]. 

According to Dieck et al., the anti-cholesterolemic activity of probiotics can be caused by, e.g., bile salt hydrolysis (BSH), interference with hepatic de novo synthesis of lipids via modulation of SCFA or satiety hormones [16]. The bile salt hydrolysis activity of some bacterial strains lowers the enterohepatic cycling of bile salt conjugates, restored by de novo synthesis from cholesterol in the liver, resulting in reduced plasma cholesterol and lipoprotein levels [16]. 

Effects of Probiotics on Lipid Levels in Patients With Metabolic Syndrome

According to a study published by Bernini et al., no statistically significant differences were observed intra- or intergroup for HDL (high-density lipoprotein) [17]. The probiotic group showed a remarkable reduction in BMI, TC, and LDL compared with baseline values. Regarding intergroup changes, there was a significant decrease (P < 0.05) in BMI, TC, and LDL verified after 45 days in the probiotic group compared with the control group [17]. The control group had a significant increase in BMI and HDL and a significant decrease in TC and LDL compared with the baseline values of the probiotic group (P < 0.05) [17].

Table 3 shows changes in cholesterol levels in control and probiotic groups of patients with metabolic syndrome after probiotic intervention [17].

**Table 3:** Changes in cholesterol levels in control and probiotic groups with metabolic syndrome after probiotic intervention. TC: total cholesterol, HDL: high-density cholesterol, LDL: low-density cholesterol Bernini et al. [17]

Parameters	Control (n = 25)		P-value	Probiotic(n=26)		P-value
	T0	T45		T0	T45	
TC (mg/dL)	199(166.5-208.5)	205 (173-220.5)	0.506	209 (183.8- 249.8)	194 (168-251)	0.009
HDL (mg/dL)	42 (35-47.5)	39 (35.5-47)	0.617	40.5 (30- 49.5)	38.5 (31.3-46)	0.820
LDL (mg/dL)	117 (83-142)	115 (95-146)	0.820	128.5 (103- 152.8)	111 (93.6- 134.6)	0.008

Another study by He et al. included the evaluation of a group of people with mild to moderate hypercholesterolemia and showed that after intake of yoghurt (which was fermented with a starter containing Bifidobacterium lactis and L. acidophilus along with bacteria in standard yoghurt) for six weeks, there was a considerable reduction in blood cholesterol [12]. Their studies showed that the intake of yoghurt containing microencapsulated bile salt hydrolase-active Lactobacillus reuteri NCIMB 30242, taken two times per day for six weeks, reduced LDL-cholesterol, total cholesterol and non-HDL cholesterol in hypercholesterolemia adults [12]. This treatment appeared to be superior to traditional probiotic therapy [12]. Their study showed that daily consumption of 200 g of fermented milk (FM) containing L. acidophilus L1 for three weeks showed a 2.4% (P < 0.05) decreased serum cholesterol concentration in comparison to the placebo group [12]. They also showed that a mixture of organisms (a probiotic mixture) comprised of Streptococcus, Saccharomyces, Bacillus, Clostridium, Candida and Lactobacillus reduced total cholesterol and liver cholesterol compared to individual bacteria strains [12]. The supplied mixed-bacteria and L. acidophilus groups displayed a 23-57% reduced cholesterol concentration in the liver [12]. In addition, the total serum cholesterol in the supplied mixed-bacteria group was decreased by 15-33% compared with that in the single-bacteria-supplemented groups [12].

A study by Pan et al. provided substantial proof that probiotic therapy application significantly reduces the risk indicators in metabolic syndrome, including TC, HDL, LDL, and WC [3]. Pooled estimates demonstrated that treatment with probiotic therapy significantly reduced the TC levels (SMD = −0.36, 95% CI −0.55, −0.17), LDL (SMD = −0.42, 95% CI −0.61, −0.22), but increased the HDL (SMD = 0.28, 95% CI.03, 0.52) [3].

A study by Cicero et al. on elderly patients over a two-month clinical trial treatment with a probiotic formula of L. plantarum PBS067-DSM 24,937, L. acidophilus PBS066-DSM 24,936 and L. reuteri PBS072- DSM 25,175 showed a decrease in LDL, HDL and TC [23]. It also decreased metabolic syndrome prevalence, several cardiovascular risk factors and markers of insulin resistance [23].

According to a study published by Arabi et al., probiotic administration significantly affected the cardiometabolic and anthropometric indices by reducing TC and LDL and increasing HDL levels [2]. Despite these favourable effects, our findings demonstrated that the desired effect of probiotics in reducing LDL was higher than the minimal clinically significant difference (MCID) for LDL (3.87 mg/dl), which is essential for clinicians to reach treatment goals [2]. The efficacy of the probiotic intervention was lower in comparison with antihyperlipidemic and anti-obesity drugs [2]. However, these changes are commensurate with lifestyle changes and can help reduce the risk of cardiovascular disease and mortality [2]. Based on previous studies, weight loss equal to or more than 5% could improve lipid profile abnormalities related to the incidence of coronary heart disease [2].

Dieck et al. showed that total cholesterol and LDL cholesterol levels decreased significantly between baseline and the end of the intervention with probiotic therapy, as shown in Table 4 [16]. In contrast, a slight increase was observed for HDL cholesterol resulting in a significant reduction in LDL/HDL ratio after the four-week intervention (p = 0.0022) [16]. 

**Table 4:** Showing reduction in cholesterol levels between baseline and end of intervention. LDL: low-density cholesterol, HDL: high-density cholesterol Dieck et al. [16]

Variables	Baseline	2 Weeks	4 Weeks	P value
	Mean (95 % CI)	Mean (95% CI)	Mean (95% CI)	
Total cholesterol (mg/dL)	179.3 (164.5 – 194.5)	172.4 (157.5- 187.4)	169.10 (155.5 – 182.7)	0.0037
LDL cholesterol (mg/dL)	120.6 (103.4- 137.7)	117.8 (100.3 – 135.3)	113.10 ((95.0–131.2)	0.0313
HDL cholesterol (mg/dL)	46.1 (41.9–50.2)	46.9 (42.7–51.2)	47.3 (42.6–52.1)	

Safety of Probiotics

The safety of the probiotic strains was tested using a cytotoxicity test, assessment of antimicrobial susceptibility, and genome analyses for Bacillus strains. No safety risk was detected. No safety issues were found by monitoring the blood routine parameters and vital signs [2,16]. Probiotic-containing supplements must be prescribed with caution in immunocompromised patients, such as those with acute pancreatitis, short bowel syndrome, acquired immunodeficiency syndrome (AIDS), lymphoma and long-term corticosteroid intake [2].

Limitations

Many of the included studies have not mentioned the dose of the probiotic and the duration it has to be taken to show its effects. The only articles selected for screening were in English and published during 2002-2022. This could have led to missing out on some relevant studies.

## Conclusions

The main objective of this article is to study the effect of probiotics in reducing cholesterol levels, particularly in patients with metabolic syndrome. The included studies have shown success in reducing blood cholesterol levels, with the exception of HDL which showed an increase. Probiotics are safe and are associated with improvement in biomarkers in metabolic syndrome. To further prove the efficacy of probiotics on cholesterol levels, research should be conducted on a large scale using randomised control trials with double or triple blinding in patients with hyperlipidaemia or metabolic syndrome.
